# Calibration of myocardial iron concentration against T2-star Cardiovascular Magnetic Resonance

**DOI:** 10.1186/1532-429X-11-S1-P224

**Published:** 2009-01-28

**Authors:** John-Paul Carpenter, Taigang He, Paul Kirk, Lisa J Anderson, John B Porter, John Wood, Renzo Galanello, Gianluca Forni, Gualtiero Catani, Suthat Fucharoen, Adam Fleming, Mike House, Greg Black, David N Firmin, Timothy G St Pierre, Dudley J Pennell

**Affiliations:** 1 Royal Brompton and Harefield NHS Trust, London, UK; 2 St George's Hospital NHS Trust, London, UK; 3 University College Hospitals NHS Trust, London, UK; 4 Children's Hospital, Los Angeles, Los Angeles, CA USA; 5 Ospedale Regionale Microcitemie, Cagliari, Italy; 6 Ospedali Galliera di Genova, Genoa, Italy; 7 Mahidol University, Puttamonthon Nakornpathom, Thailand; 8 The University of Western Australia, Perth, Australia

**Keywords:** Cardiovascular Magnetic Resonance, Iron Concentration, Ventricular Free Wall, Siderosis, Myocardial Iron

## Introduction

Heart failure secondary to myocardial siderosis remains a major problem for patients with transfusional iron overload. Direct measurement of myocardial iron concentration by biopsy is not only highly invasive but unreliable due to uneven myocardial iron deposition. T2*cardiovascular magnetic resonance (CMR) is the only non-invasive technique which is able to assess tissue iron levels. This is a rapid, robust technique with proven reproducibility and inter-site transferability which has been accepted as part of clinical practice worldwide [1]. However, little data is available on the calibration of T2* values against tissue concentration of iron [2].

## Purpose

The aim of this study was to calibrate T2* cardiovascular measurements versus mycardial iron concentration.

## Methods

With full ethical approval, seven whole-hearts were donated from patients with transfusion-dependent anaemia. Six of the hearts were from patients with end-stage heart failure. The seventh was from a patient who died from cerebrovascular disease and had no symptoms of heart failure prior to death. 4 hearts were post-mortem specimens and 3 hearts were from patients who had cardiac transplantation for end-stage heart failure.

All hearts were formalin fixed before being sliced into 5 or 6 short axis slices (depending on the size of the organ). The apical slice was not used for analysis. A custom-made Perspex plinth was used to hold each of the slices which were then scanned immersed in water at 37°C using a 1.5 T Avanto MR scanner (Siemens, Erlangen, Germany) with a four-channel phased-array coil. A multi-echo T2-star sequence (gradient echo) was used: TE = 2.47, 4.48, 6.49, 8.5, 10.6, 12.7, 14.8, 16.9 ms; field of view 150 × 150 mm; matrix 128 × 128; flip angle 35°; number of excitations 2; bandwidth 815 Hz per pixel; TR 20 ms; slice thickness 5 mm.

**Figure 1:**
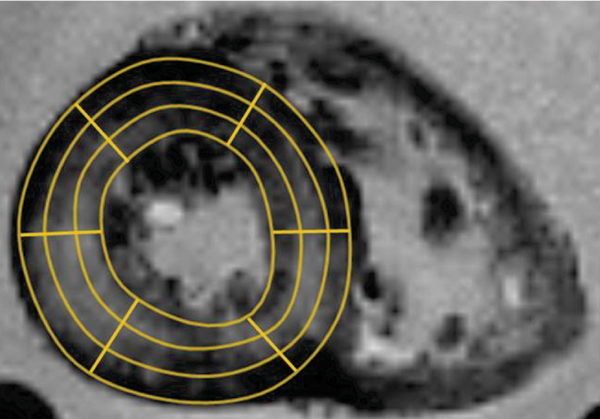
MR T2* image showing cardiac short axis slice divided into regions of interest for T2* analysis.

Each of the slices was subsequently divided into 6 sectors, each sector being subdivided into epicardial (outer), mesocardial (mid) and endocardial (inner) myocardial layers. Two specimens were taken from the right ventricular free wall. Analysis of myocardial iron concentration was performed using inductively coupled atomic emission spectrometry (after digestion in acid to avoid sampling errors). T2* was measured using Thalassaemia tools (a plug-in of CMRtools, Cardiovascular Imaging Solutions, London) in the corresponding regions of myocardium (see Figure 1). A truncation method was used for curve-fitting as previously described [3].

## Results

**Figure 2:**
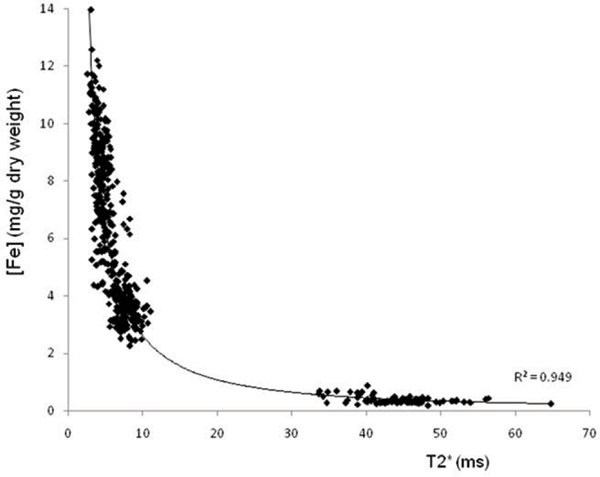
**Graph of myocardial iron concentration (mg Fe/g dry weight) vs. T2-star (ms)**. R^2^ = 0.949.

**Figure 3:**
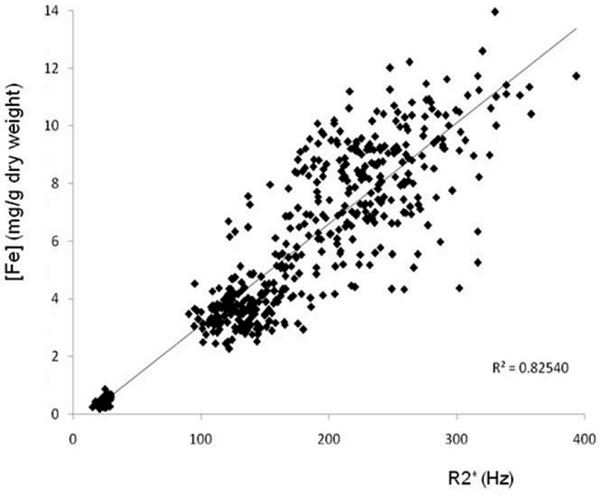
**Graph of myocardial iron concentration (mg Fe/g dry weight) vs R**^2^* (Hz). R^2^ = 0.825

31 short axis myocardial slices from the 7 hearts were analysed. A total of 558 specimen blocks (mean mass +/- SD = 1001 +/- 620 mg) and the corresponding region of interest (ROI) on CMR were included in the analysis. 21 (3.6%) of the ROIs were excluded from the final analysis due to technical issues (such as artefact affecting T2-star analysis). The mean wet (dry) tissue iron concentration ranged from 0.07 (0.35) to 1.86 (8.08) mg Fe/g. T2-star values ranged from 2.54 to 64.7 ms. Iron concentration was related to T2* and R2* (Figures 2 and 3).

## Conclusion

There is a linear relationship between tissue iron concentration and R2* and a non-linear relationship with T2*. The results accord with published data on myocardial iron concentration in non-iron loaded hearts, and a previous report in a single iron loaded post-mortem heart [2, 4]. *In vivo* T2* correlates well with that of ex-vivo formalin-fixed tissue, and therefore these data form a calibration curve for myocardial iron concentration over a wide range from normal to fatal levels [5].
